# 13-Tetradecenyl acetate, a female-produced sex pheromone component of the economically important click beetle *Melanotus communis* (Gyllenhal) (Coleoptera: Elateridae)

**DOI:** 10.1038/s41598-019-52199-1

**Published:** 2019-11-07

**Authors:** Livy Williams, Jacqueline M. Serrano, Paul J. Johnson, Jocelyn G. Millar

**Affiliations:** 4 Insect Biodiversity Laboratory, South Dakota State University, Brookings, SD 57007 USA; 1 USDA-ARS U.S. Vegetable Laboratory, Charleston, SC 29414 USA; 2 Department of Entomology, University of California, Riverside, CA 92521 USA; 3 USDA-ARS Temperate Tree Fruit and Vegetable Research Laboratory, Wapato, WA 98951 USA

**Keywords:** Zoology, Ecology

## Abstract

Species-specific behavior-modifying chemicals have been used for more than 50 years for monitoring and management of insect pests of agriculture and human health. Elaterid beetle larvae are among insect pests in soil that are increasingly problematic, in part due to the lack of effective management strategies. However, little is known about the insect-produced chemicals that mediate the reproductive behavior of these pests. We used chemical and behavioral studies to identify, synthesize, and field test the sex attractant pheromone of adults of *Melanotus communis*, commonly called the corn wireworm, the larvae of which are economically important pests of U.S. crops. Our results indicated that a single female-produced chemical, 13-tetradecenyl acetate, was strongly attractive to conspecific male beetles, and did not appear to attract other species. In field evaluations, male *M. communis* exhibited a dose-dependent response to this compound. In a trial comparing different slow-release dispensers, a small rubber septum impregnated with the chemical was as effective as and easier to use than a plastic bag dispenser. Given that the sex attractant of this insect consists of a single compound that can be readily synthesized, its development for monitoring and management of the corn wireworm may be economically feasible.

## Introduction

The click beetles (Coleoptera: Elateridae) comprise a large and diverse family of insects, with > 12,000 described species worldwide (P.J. Johnson, unpubl. catalog). The larvae of pest species are known as wireworms, and can be significant agricultural pests. Their importance as pests is increasing due to the phasing out of effective but environmentally detrimental insecticides that had been used in their control over the past several decades. Sex pheromones or sex attractants have been developed for monitoring and management of a number of Eurasian click beetle species^1^. However, to date, pheromones have been identified from only two North American species, in the genus *Cardiophorus* Eschscholtz^2^, from the ~1,000 known species on this continent^3,4^. Earlier reports of pheromone identifications for the North American species *Limonius californicus* (Mannerheim)^5^ and *L. canus* LeConte^6,7^ have not been substantiated in subsequent field trials (unpublished data; JMS in California, P. Landolt and D. Horton in Washington state, G. Reddy in Montana).

In the eastern United States and southeastern Canada, the genus *Melanotus* Eschscholtz includes several economically important species, including *M. communis* (Gyllenhal), *M. depressus* (Melsheimer), and *M. verberans* (LeConte). In particular, *M. communis* is an important pest of maize, small grains, sugarcane, and vegetable crops in much of the United States east of the Rocky Mountains^8–12^. Effective insecticidal options for in-season control of wireworms are disappearing, with currently registered insecticides being of limited efficacy^13,14^. There is some promise with sugarcane genotype resistance^15^ and some non-lethal methods could help to manage wireworms in sugarcane^16–19^. However, even low populations of *M. communis* are a serious concern to sugarcane growers because it has been reported that just one wireworm per 1.5 m of sugarcane row can cause a reduction in stand and yield by 7.0% and 3.8%, respectively^10^. There is also a low market tolerance for injury to tuber/root vegetables, such as potatoes and sweetpotatoes, due to the feeding holes caused by wireworms. These feeding holes can also facilitate attack by other invertebrates as well as pathogens. Thus, additional methods of monitoring and managing *M. communis* would be an asset for multiple cropping systems. One such method that might be explored is the development of practical applications for sex pheromones of this species, as has been done for a congener^20,21^.

Pheromone glands of female *M. fusciceps* (Gyllenhal) and *M. castanipes* (Paykull) from western Russia contained complex mixtures that included esters of 14-carbon saturated and unsaturated alcohols, but the exact structures and their evaluation in field trials have not been reported^22^. In contrast, research groups in Japan identified and field-tested female-produced sex pheromones for *M. okinawensis* Ôhira (dodecyl acetate)^1,23^ and *M. sakishimensis* Ôhira ((*E*)-9,11-dodecadienyl butyrate and (*E*)-9,11-dodecadienyl hexanoate)^1,24^. (*E*)-9,11-Dodecadienyl butyrate and (*E*)-9,11-dodecadienyl hexanoate have also been identified in extracts from *M. tamsuyensis* Bates, but field trials with the two compounds were not reported^25^. Practical applications for these pheromones have since been developed for monitoring purposes, and for control via mass trapping and mating disruption^20,21,26–30^.

The serendipitous discovery that male *M. depressus* were attracted to (*E*)-11-tetradecenyl acetate and (*E*)-11-tetradecenol^29,30^, the pheromone components of the tufted apple bud moth (TABM), *Platynota idaeusalis* (Walker)^31^, suggested that one or both of these compounds might also be pheromone components of *Melanotus* spp. However, to our knowledge, it was never determined whether *M. depressus* females actually produced (*E*)-11-tetradecenyl acetate and/or (*E*)-11-tetradecenol. This fragmentary data from several *Melanotus* spp. from North America and Eurasia suggested that the pheromones of species in this genus are likely to be esters of saturated or unsaturated 12- or 14-carbon alcohols.

Thus, the goal of the research reported here was to identify the sex pheromones of one or more North American *Melanotus* species, with the working hypothesis that the identification of the pheromone for one species would likely provide insight into the pheromones of congeners. As a first model species, we focused our attention on the economically important *M. communis*. Here, we report the identification, synthesis, and field-testing of 13-tetradecenyl acetate and several related compounds as possible female-produced sex pheromone components for this species.

## Methods

### Insect collection and rearing

*Melanotus communis* adults were collected from agricultural fields at the USDA-ARS U.S. Vegetable Laboratory and Clemson University Coastal Research and Extension Center in Charleston, SC, USA, (32°44′44.06″N 80°03′40.35″W, elev. 4 m) intermittently from May to September in 2016 and 2017. Beetles were collected with Malaise traps, blacklight traps, at incandescent lights, and by hand under debris and vegetation in the fields. Malaise traps were used without a killing agent, and were monitored several times daily to collect live beetles. Light traps were monitored at night and again shortly after sunrise. Mating status, age, and other life history factors of the beetles were not known. After collection, adults were held individually in ventilated transparent 45 ml plastic vials (12 dram, No. 55–12, Thornton Plastics Co., Salt Lake City, UT, USA) streaked with honey and containing a piece of paper towel moistened with distilled water. Insects were held (22 °C ± 1, 50% r.h., L:D 16:8) for 1 to 7 d prior to overnight shipment to the University of California, Riverside Entomology Quarantine Facility (USDA-APHIS permits P526P-14-03526 and P526P-17-02384). All *Melanotus* species were identified by comparison with reference specimens of known identity, supported with genital morphology and taxonomic keys^32^. Voucher specimens are deposited in the Severin-McDaniel Insect Research Collection at South Dakota State University, the Entomology Research Museum at the University of California, Riverside, and the National Museum of Natural History through the USDA-ARS Systematic Entomology Laboratory, Washington, D.C.

### Preparation and analysis of extracts of potential female-produced pheromones

Headspace volatiles were collected from individual live females held in 250 ml glass canning jars, with lids cut from Teflon^TM^ sheet (ca. 1.6 mm thickness; McMaster-Carr, Elmhurst, IL, USA) and fitted with an air inlet and outlet. The beetles were provided with 10% sugar water for nutrition (in a glass vial with a cotton-tipped applicator wick) and a small wire mesh perch. Emitted compounds were trapped on collectors made from a glass tube (0.5 cm ID) with a 1-cm-long bed of activated charcoal (50–200 mesh; Fisher Scientific, Pittsburgh, PA, USA), held in place by glass wool plugs. For the female that was shipped to UC Riverside in June 2016, humidified and charcoal purified air was pushed through the glass jar at a flow rate of 250 ml/min for 4 d. In contrast, for collection of headspace volatiles from two females that were shipped to UC Riverside in June 2017, charcoal filtered air was pulled through the jars at 250 ml/min by applying a slight vacuum to the outlet. Headspace volatiles were collected for 3 d. All collections were conducted in a temperature controlled room (23 °C), equipped with fluorescent lighting and a north-facing window (~5 m × 1 m) that provided ambient lighting (ca. 12:12 L:D). Trapped volatiles were eluted from the collectors with 0.5 ml of dichloromethane (Optima grade, Fisher Scientific, Pittsburg, PA, USA). After collections of headspace volatiles were terminated, volatiles were collected from abdomens of freeze-killed females. Individual abdomens were crushed in a 20 ml glass vial, the vial was sealed with aluminum foil, and the volatiles were sampled using solid phase microextraction (SPME)^2^. Volatiles were absorbed for 2 h onto a SPME fiber pushed through the aluminum foil sealing the vial (100 μm PDMS; Supelco, Bellefonte, PA, USA), then analyzed as described below.

Extracts of headspace volatiles were analyzed by coupled gas chromatography-mass spectrometry (GC-MS) with an Agilent 7820 A gas chromatograph coupled to an Agilent 5977E mass selective detector (Agilent Technologies, Santa Clara, CA, USA) fitted with an autosampler and an HP-5 column (30 m × 0.25 mm ID, Agilent). Injections were made in splitless mode, with the purge valve opened after 30 sec. The oven was programmed from 40 °C for 1 min, then increased 10 °C/min to 280 °C. Volatiles from crushed abdomens that were adsorbed onto SPME fibers were analyzed with an Agilent 6890 N gas chromatograph interfaced to an Agilent 5975 C mass selective detector. The GC was also fitted with a HP-5 column and the temperature program used was 40 °C for 1 min, then increased 10 °C/min to 280 °C, hold for 20 min. Loaded SPME fibers were desorbed in the injector port (250 °C) in splitless mode for 30 sec prior to starting the run. Compounds were tentatively identified by mass spectral interpretation, matches with database spectra (W8N05ST; Wiley version 8.0 and NIST, version 5.0), and matches with published retention indices^33^. Identifications were confirmed by matching retention times and mass spectra with those of authentic standards.

Coupled gas chromatography-electroantennogram detection (GC-EAD) was used to test the responses of the antennae of male *M. communis* beetles to various C14 acetates, specifically tetradecyl acetate, 13-tetradecenyl acetate, (*E*)-11-tetradecenyl acetate, and (*E*)-11,13-tetradecadienyl acetate. For all males, the legs were removed and genitalia were glued shut with Super Glue^®^ before they were taken out of the quarantine facility (a requirement of the permit) for use in GC-EAD analyses. Analyses were conducted on an HP 5890 Series II GC (Hewlett-Packard, now Agilent) fitted with a DB-WAX column (30 m × 0.25 mm ID × 0.25 μm film; J&W Scientific, Folsom, CA, USA). The effluent from the column was split using an ‘X’ cross, with half of the sample going to the flame-ionization detector and the other half to the antennal preparation, with the 4^th^ arm of the X-cross providing make-up gas (~0.62 ml/min). The portion directed to the EAD was diluted in a humidified air stream (~550 ml/min) directed over the antennal mounting block^2^. Because the four acetates have similar retention times, GC-EAD analyses of the standards were done by alternating injections of the four compounds at ~2.5 min intervals using an isothermal oven temperature (220 °C) in split mode (injector temperature 250 °C), so that an antennal preparation was exposed sequentially to each synthetic compound during a run (n = 12 replicates, using one antenna each from four males).

### Authentic standards of pheromone candidates

Dodecyl acetate (henceforth 12:OAc) was purchased from TCI America (Portland, OR, USA), and 11-dodecenyl acetate (henceforth 11–12:OAc) and (*E*)-11-tetradecenyl acetate (henceforth *E*11-14:OAc) from Bedoukian Research (Danbury, CT, USA). Tufted apple bud moth sex pheromone lures were purchased from Scentry Biologicals Inc. (Billings, MT, USA).

Other compounds were prepared as described below. Unless otherwise specified, solutions were dried over anhydrous Na_2_SO_4_, and concentrated by rotary evaporation under partial vacuum. Liquid chromatography purifications were carried out with 230–400 mesh silica gel. Tetrahydrofuran (THF) was purified by distillation from sodium benzophenone ketyl, and all reactions with air- or water-sensitive reagents were carried out in oven-dried glassware under argon.

### Synthesis of tetradecyl acetate (henceforth 14:OAc)

Tetradecyl acetate was prepared by dropwise addition of acetyl chloride (2.14 ml, 30 mmol) to an ice-bath cooled solution of tetradecanol (5.35 g, 25 mmol), pyridine (2.42 ml, 30 mmol), and dimethylaminopyridine (~100 mg) in 100 ml CH_2_Cl_2_. The mixture was then warmed to room temperature and stirred for 3 h, followed by addition of 1 ml EtOH and stirring for 1 h, to eliminate excess acetyl chloride as ethyl acetate. The mixture was then concentrated, and the residue was partitioned between hexane and water. The hexane layer was washed sequentially with aqueous 1 M HCl, saturated aq. NaHCO_3_, and brine, then dried and concentrated. The residue was purified by Kugelrohr distillation (oven temp ~105 °C; 0.15 mm Hg), yielding the ester as a clear, colorless liquid (5.60 g, 88%). ^1^H NMR (400 MHz, CDCl_3_): δ 4.03 (t, 2H, J = 6.8 Hz), 2.03 (s, 3H), 1.56–1.64 (m, 2H), 1.37–1.20 (m, 22 H), 0.87 (t, 3H, J = 6.4 Hz). ^13^C NMR (100 MHz, CDCl_3_): δ 171.20, 64.64, 31.92, 29.65, 29.57, 29.52, 29.35, 29.26, 28.60, 25.91, 22.68, 20.98, 14.09. MS (*m/z*, %): 241 (M^+^ -15, trace), 196 (3), 184 (trace), 168 (4), 154 (1), 153 (1), 140 (2), 139 (2), 130 (trace), 125 (7), 116 (4), 111 (19), 97 (39), 83 (52), 69 (51), 61 (41), 55 (53), 43 (100).

### Synthesis of 13-tetradecenyl acetate (henceforth 13–14:OAc)

A solution of 11-bromoundecanol (5.02 g, 20 mmol), dihydropyran (2.26 ml, 25 mmol), and ~50 mg *p*-toluenesulphonic acid (PTSA) in 50 ml ether was stirred overnight at room temperature. The mixture was then diluted with hexane, extracted twice with saturated aq. NaHCO_3_ and once with brine, then dried and concentrated. The residue was purified by vacuum flash chromatography, eluting with 5% EtOAc in hexane, yielding 6.66 g of the THP-protected bromoalcohol (99%). This compound was taken up in 30 ml dry THF, and ~10 ml of the solution was added to a dry, argon-flushed flask charged with 0.72 g of freshly ground Mg chips. Once the Grignard reaction had started, as evidenced by warming of the flask and a grey color, the remainder of the solution was added to the flask over ~30 min. The mixture was then warmed to 50 °C and stirred for 1.5 h, then cooled to room temperature.

An oven-dried flask flushed with argon was charged with 300 mg CuI and 40 ml dry THF, and cooled to ~−10 °C in an ice-acetone bath. The Grignard solution was then added dropwise over 40 min with a syringe pump, producing a blue-black slurry. Allyl bromide (2.42 g, 20 mmol) in 5 ml THF was then added dropwise by syringe pump over 45 min, and the resulting mixture was stirred an additional 30 min, then quenched with saturated aqueous NH_4_Cl, and diluted with hexane. The organic layer was washed with saturated aqueous NH_4_Cl and brine, then dried and concentrated. The residue was taken up in 100 ml MeOH, ~50 mg of PTSA was added, and the mixture was stirred overnight at room temperature. Then, 5 g of solid NaHCO_3_ were added, and most of the MeOH was removed by rotary evaporation. The residue was partitioned between hexane and water, and the hexane layer was washed with saturated aqueous NaHCO_3_ and brine, then dried and concentrated. The crude product was purified by vacuum flash chromatography, eluting with 25% EtOAc in hexanes, followed by Kugelrohr distillation (oven temp ~80 °C, 0.05 mm Hg), yielding 3.33 g (15.7 mmol, 79%) of 13-tetradecenol. The alcohol was taken up in CH_2_Cl_2_, pyridine (1.58 g, 20 mmol) and dimethylaminopyridine (~100 mg) were added, and the solution was cooled in an ice-bath. Acetyl chloride (1.44 ml, 20 mmol) was added in one aliquot, and the mixture was warmed to room temperature and stirred for 3 h. The reaction was not complete, so a further 1 ml of pyridine and 0.5 ml of acetyl chloride were added, and the mixture was stirred an additional 2 h. EtOH (1.5 ml) was then added, and the mixture was stirred 1 h. The mixture was then concentrated, and the residue was partitioned between hexane and water. The hexane layer was washed sequentially with aqueous 1 M HCl, saturated aqueous NaHCO_3_, and brine, then dried and concentrated. The crude product was purified by Kugelrohr distillation, first removing a forerun fraction (oven temp < 80 °C at 0.05 mm Hg, ~0.5 g), followed by distillation of the desired product (oven temperature ~90 °C, 0.04 mm Hg), yielding 13-tetradecen-1-yl acetate (2.81 g, 70%), which gave a single peak on GC analysis. ^1^H NMR (400 MHz, CDCl_3_): δ 5.80 (m, 1 H), 4.98 (br d, J = 14.1 Hz, 1 H), 4.92 (br d, J = 10.2 Hz, 1 H), 4.04 (t, J = 6.8 Hz, 2 H), 2.07–1.98 (m, 5 H), 1.64–1.56 (m, 2 H), 1.43–1.22 (m, 18 H). ^13^C NMR (400 MHz, CDCl_3_): δ 171.19, 139.21, 114.08, 64.64, 33.81, 29.59, 29.55, 29.50, 29.25, 29.14, 28.94, 28.60, 25.91, 20.99. MS (*m/z*, %): 194 (M^+^-AcOH, 5), 166 (3), 152 (2), 151 (2), 138 (5), 137 (5), 123 (10), 109 (21), 105 (1), 96 (55), 91 (2), 82 (83), 77 (3), 68 (75), 61 (16), 55 (100), 51 (1), 43 (90).

### Synthesis of (*E*)-11,13-tetradecadien-1-yl acetate (henceforth E11,13–14:OAc)

A solution of THP-protected 11-dodecyn-1-ol (4.2 g, 16 mmol) and ~50 mg triphenylmethane indicator in dry THF was cooled to ~−10 °C in an ice-salt bath and stirred under argon. A solution of BuLi (8 ml, 20 mmol, 2.5 M in hexanes) was added dropwise, resulting in a bright red solution. Dry paraformaldehyde (4 g) was then added in one portion, and the mixture was warmed to room temperature overnight. The mixture was quenched with saturated aqueous NH_4_Cl and extracted sequentially with hexane and ether. The combined organic layers were washed with water and brine, dried, and concentrated to a colorless oil which was added dropwise to a slurry of LiAlH_4_ (1.32 g, 35 mmol) in 60 ml dry THF at 0 °C under argon. The mixture was heated to 65 °C for 2 h, then cooled in an ice-bath and quenched by dropwise sequential addition of 1.4 ml water, 1.05 ml 20% aqueous NaOH, and 4.9 ml water. The resulting slurry was stirred overnight, then filtered through a pad of Celite^®^, rinsing with ether. The filtrate was dried and concentrated, and the residue was purified by vacuum flash chromatography, eluting with 20% EtOAc in hexanes, yielding 2.75 g of the desired alkenol product as one peak by GC analysis. The purified alcohol was taken up in 150 ml CH_2_Cl_2_ and the solution was cooled in an ice-bath, then activated MnO_2_ was added (29 g, 333 mmol), and the mixture was warmed to room temperature and stirred overnight. The mixture was filtered through a pad of Celite^®^, rinsing well with CH_2_Cl_2_. After concentration, the crude aldehyde was used directly in the next step.

A dry flask was charged with methyltriphenylphosphonium bromide (5.54 g, 15.5 mmol) and dry THF (100 ml). The slurry was cooled in an ice-bath, and potassium *t*-butoxide (1.68 g, 15 mmol) was added in one portion. The mixture was stirred at 0 °C for 15 min, then at room temperature for 3 h, yielding a milky yellow slurry. The slurry was cooled to 0 °C, and a solution of the crude aldehyde (1.9 g, ~6.4 mmol) in 10 ml THF was added by syringe pump over 1 h. The resulting mixture was slowly warmed to room temperature over 3 h, then quenched with saturated aqueous NH_4_Cl. The mixture was extracted with hexane, and the hexane layer was washed with brine, dried, and concentrated. The residue was taken up in 50 ml hexane to precipitate most of the triphenylphosphine oxide byproduct, and the resulting slurry was filtered. The filtrate was concentrated, the residue was taken up in 25 ml MeOH, and ~50 mg PTSA was added. The solution was stirred for 3 h at room temperature, then 2 g solid NaHCO_3_ were added, and the mixture was concentrated. The residue was partitioned between hexane and water, and the hexane layer was washed sequentially with water and brine, then dried and concentrated and purified by vacuum flash chromatography, eluting with 25% EtOAc in hexane, yielding the dienol (1.03 g, 77%).

The dienol (0.53 g, 2.5 mmol) was acetylated as described above. The crude product was purified by Kugelrohr distillation (oven temp ~85 °C, 0.05 mm Hg), yielding (*E*)-11,13-tetradecadien-1-yl acetate as a colorless oil (0.52 g, 82%), 97% pure by GC. ^1^H NMR (400 MHz, CDCl_3_): δ 6.30 (ddd, J = 17.0, ~10.3 Hz, ~10.3 Hz, 1 H), 6.04 (br dd, J = 15.2, 10.4 Hz, 1 H), 5.70 (dt, J = 15.2, 6.9 Hz, 1 H), 5.07 (br d, J ~ 17.5 Hz, 1 H), 4.94 (br d, J ~ 10.2 Hz, 1 H), 4.04 (t, J = 6.8 Hz, 2 H), 2.10–2.02 (m, 2 H), 2.04 (s, 3 H), 1.64–1.58 (m, 2 H), 1.42–1.24 (m, 14 H). ^13^C NMR (400 MHz, CDCl_3_): δ 171.24, 137.37, 135.59, 130.86, 114.57, 64.66, 32.55, 29.48, 29.46, 29.24, 29.18, 28.60, 25.90, 21.02. MS (*m/z*, %): 252 (M^+^, 5), 209 (1), 192 (8), 177 (trace), 163 (4), 149 (7), 121 (23), 110 (16), 107 (16), 95 (39), 81 (71), 79 (71), 73 (3), 67 (100), 61 (9), 54 (53), 43 (92).

### Field bioassays of synthetic pheromone candidates

All field bioassays were conducted at the USDA-ARS U.S. Vegetable Laboratory and Clemson University Coastal Research and Extension Center in Charleston, SC. This shared facility encompasses a total of ca. 295 ha, of which ca. 95 ha are crop fields, with the remainder being buildings, wooded areas, and wetlands. In 2016, we tested pheromone candidates for *M. communis*, including the compounds identified from extracts of the insects, and several related compounds (Table 1). The main focus of this field trial was to determine the activity of the insect-produced 14:OAc and 13–14:OAc on *M. communis* males. In addition, the 12-carbon acetates were included due to their structural similarities to the pheromone components of *M. okinawensis*^23^. *E*11-14:OH, *E*11-14:OAc, and *E*11,13-14:OAc were tested to determine if they were attractive or synergized attraction to 13–14:OAc and 14:OAc, and *E*11,13–14:OAc was tested due to its structural similarities to pheromone components identified from *M. sakishimensis* and *M. tamsuyensis*^24,25^. The sex pheromone of the tufted apple bud moth (TABM), *Platynota idaeusalis*, (2:1 ratio of (*E*)11-14:OAc + (*E*)11-14:OH) was included because of reports^29,30^ of attraction of *Melanotus* spp. to TABM lures. Field trials were conducted for 6 wk (4 August – 13 September 2016) at three fields; one planted to maize (0.52 ha), one to sweetpotato (1.13 ha), and the other to cucumber (0.69 ha). Distance between these three fields ranged from 360 to 570 m. For all treatments (with the exception of the commercial TABM lure), ~10 mg of undiluted blend was pipetted into a 0.2 ml PCR tube (Multiply-Pro; Sarstedt AG & Co., Nümbrecht, Germany) with a pinhole in the lid made with a no. 3 insect pin. Empty, punctured PCR tubes were used as controls. Lures were suspended from the center of each trap. Lures were replaced and treatment locations in each transect were re-randomized biweekly. Linear trap transects were established at each field. In each transect, seven Pherocon^®^ 1 C wing traps (white, ca. 27 × 23 cm; Trécé Inc., Adair, OK, USA) were deployed at the border between the field and an adjacent woodlot. Traps were spaced 12 m apart, and were hung 1 m above the ground from wooden stakes. Click beetles were removed from traps weekly and the adhesive was removed with xylene and an ultrasonic cleaner^34^.

**Table 1:** Compounds and blends used for field bioassays of potential pheromone components of *Melanotus communis* in 2016 and 2017.

Year tested	Compound(s) and ratio
2016, 2017	14:OAc
2016, 2017	13–14:OAc
2016, 2017	1:1 blend of 14:OAc to 13–14:OAc
2016, 2017	4:1 blend of 13–14:OAc to 14:OAc*
2016	1:1 blend of 12:OAc to 11–12:OAc
2017	1:1:1:1 blend of 14:OAc to 13–14:OAc to *E*11-14:OAc to *E*11,13–14:OAc
2016, 2017	2:1 blend of *E*11-14:OH to *E*11-14:OAc^†^

^*^Natural ratio found in SPME extracts from crushed abdomens of female *M. communis* beetles.

^†^TABM lure.

In 2017 field bioassays were conducted at 11 field sites for 18 wk (1 May – 6 September) with six test treatments and a control (Table 1). These fields were planted to maize, with the exception of two fields, one of which was planted to sweetpotato, and the other was fallow. These 11 fields ranged in size from 0.32 to 0.77 ha, and were from 50 to 1210 m apart. The majority of the treatments were the same as the 2016 field trials, with the exceptions of the 12-carbon acetates and the quaternary mixture of 14-carbon acetates. *E*11-14:OAc and *E*11,13-14:OAc were added to a blend of 13–14:OAc and 14:OAc to determine if they synergized attraction to the pheromone candidates. Test blends were diluted with isopropanol ( ≥ 99.5% purity; Sigma-Aldrich, St. Louis, MO, USA) to a concentration of 5.45 mg/ml. One milliliter of each treatment blend was pipetted into 5 × 7.5 cm (~0.05 mm thickness) low-density polyethylene zipper seal bags (#01-816-1 A; Thermo-Fisher Scientific, Waltham, MA, USA), which were hung from the center of the traps. Vernon Pitfall Traps^®^ (ca. 17 × 14 cm) constructed of black polypropylene were deployed in transects in the approximate center of each field, with traps in each transect spaced 12 m apart. These traps combine the attributes of pitfall and pheromone traps, and have been successfully used to test attractants for other click beetle species^35^. The killing agent in the traps was 12 ml of a 1:1 mixture of water and propylene glycol (Prestone LowTox^®^ Antifreeze/Coolant; Prestone Products Corp., Lake Forest, IL, USA). Click beetles were removed from traps weekly and lures were replaced and treatment locations in each transect were re-randomized biweekly.

### Dose-response test of 13-tetradecenyl acetate

Having determined that 13–14:OAc was as good or better as an attractant than any blend of 14-carbon acetates (see results), we conducted field trials to determine the optimal dose of 13–14:OAc for attraction of *M. communis*. In 2018, a trial was set up for 8 wk (25 June – 20 August) in six fields; three planted to maize, and one field each to cucumber, tomato, and peanut. These six fields ranged in size from 0.38 to 0.73 ha, and were from 50 to 1135 m apart. At each field site a trap transect was established along the field border using five Vernon Pitfall Traps^®^, each 12 m apart. Test doses were 0.33, 1.0, 3.3, and 10 mg/ml of 13–14:OAc (1 ml loads), with an isopropanol control, dispensed from zipper-seal polyethylene bags as described above. Beetles were collected weekly, and lures were replaced and re-randomized every 2 wk.

### Comparison of pheromone dispensers for 13-tetradecenyl acetate

In 2018, a trial was set up for 7 wk (22 June – 10 August) to compare two lure devices at 12 fields different from those in the dose-response study described above. Eight of these fields were planted to maize, two fields to cucumber, and one field each to tomato and peanut. These 12 fields ranged in size from 0.38 to 0.73 ha, and were from 45 to 1779 m apart. At each field site, two Vernon Pitfall Traps^®^ were deployed along the field border, 12 m apart. At each site, one of the traps was baited with a zipper-seal bag (as described above) and the other with a grey rubber septum (9 mm × 19 mm, 6.2 mm ID; West Co., Lionville, PA, USA; item # 1888 grey). The zipper seal bags were loaded with 1.0 ml of a 10 mg/ml solution of 13–14:OAc in isopropanol. Rubber septa were Soxhlet-extracted with dichloromethane for 8 h, then dried in a fume hood before being loaded with 0.2 ml of a hexane solution of 13–14:OAc (50 mg/ml). Loaded septa were allowed to absorb the pheromone solution and dry, then were sealed in glass vials for shipment to South Carolina. Treatment locations were re-randomized and click beetles were collected weekly.

### Diversity of *Melanotus* species at the field sites and cross-attraction

To gain a better understanding of the *Melanotus* species occurring at our field sites, two unbaited Malaise traps (Townes style; Sante Traps, Lexington, KY, USA) were set up at two of the fields (traps separated by 620 m) at the border between the woodlot and the field. Every year (2016–2018) trapping began in April and was terminated in November. Collection vessels contained ca. 50 ml of a 1:1 mixture of water and propylene glycol as described above. Traps were serviced weekly. *Melanotus* specimens captured in these traps were identified to species as described above. Voucher specimens are deposited in the same museums noted above for the specimens collected for putative pheromone analysis.

### Statistical analysis

Captures (total catch/trap) of *M. communis* were analyzed using a multi-response permutation procedure (MRPP)^36^. MRPP is a nonparametric multivariate analysis that does not require distributional assumptions of the dependent variables. Each field was considered as a replicate in the analysis. Data were square-root transformed prior to MRPP analysis using Euclidean distance to conduct the distance matrix (PC-ORD 7.0; MjM Software Design, Gleneden Beach, OR, USA)^37^. Pairwise comparisons of treatments were Benjamini-Hochberg^38^ adjusted using a false discovery rate of 0.25. Unadjusted P and A (chance-corrected within-group agreement) values are presented in the results. Capture data from comparison of pheromone dispensers in 2018 were square-root transformed prior to paired t-test.

## Results

### Identification of pheromone candidates

**Figure 1:**
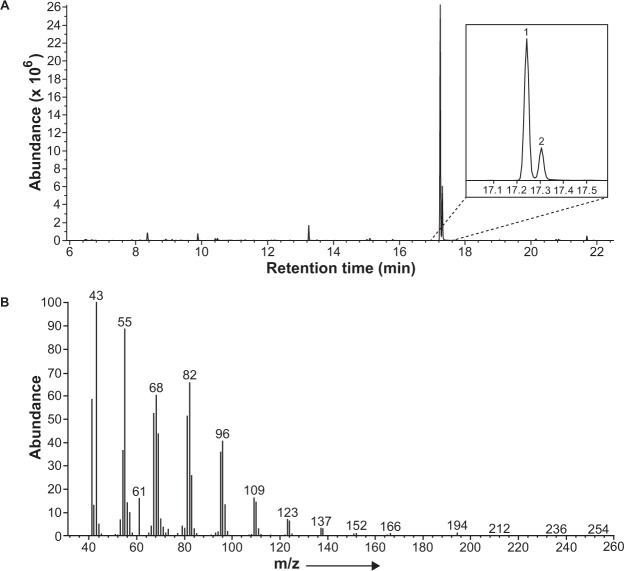
(**A**) Representative total ion chromatogram of an SPME extract of volatiles from the crushed abdomen of an adult female *Melanotus communis*, highlighting the possible pheromone candidates 13-tetradecen-1-yl acetate (peak 1) and tetradecyl acetate (peak 2); (**B**) EI mass spectrum (70 eV) of the female-produced 13-tetradecen-1-yl acetate.

Coupled GC-MS analyses of extracts of headspace volatiles collected from live female *M. communis* did not show peaks from any likely pheromone candidates, but only traces of hydrocarbons and contaminants. However, GC-MS analyses of SPME-trapped headspace volatiles from three separate crushed abdomens of these same beetles revealed two major peaks in the total ion chromatograms (Fig. 1A) which were absent in the corresponding chromatograms of extracts of headspace volatiles from the live individuals. The two compounds were tentatively identified from matches of their mass spectra with database spectra as tetradecyl acetate and a monounsaturated analog (Fig. 1B). For the unsaturated 14-carbon acetate, the double bond was tentatively determined to be in the terminal position, by comparison of its Kováts retention index with the retention indices of all possible straight-chain monounsaturated 14-carbon acetates on a DB-5 column^33^. The identifications of the two female-produced compounds were confirmed by matching their retention times and mass spectra with those of authentic standards.

**Figure 2:**
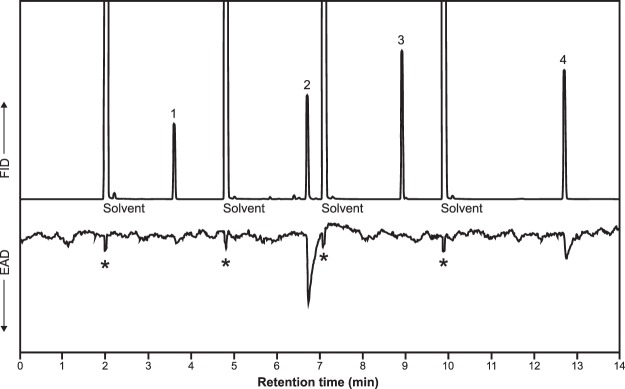
Representative GC-EAD analysis of alternating injections of 14:OAc (peak 1), 13–14:OAc (peak 2), *E*11-14:OAc (peak 3), and *E*11,13–14:OAc (peak 4), stimulating an antenna from a male *M. communis* beetle. Top trace: GC chromatogram; bottom, inverted trace: signal from antennal responses. Large peaks in the GC trace are from the solvent used to make sequential injections, and the asterisks indicate the small responses elicited from the antenna by the solvent.

Additionally, in GC-EAD analyses testing the synthesized pheromone components and analogs, antennae of male beetles were challenged sequentially with 14:OAc, 13–14:OAc, *E*11-14:OAc, and *E*11,13–14:OAc. Of the four compounds, 13–14:OAc consistently elicited the strongest responses from antennae of males (Fig. 2). There were negligible antennal responses to *E*11-14:OAc, and smaller and inconsistent responses to 14:OAc and *E*11, 13–14:OAc (Fig. 2).

## Field bioassays

### Comparison of synthetic sex pheromone candidates

**Figure 3:**
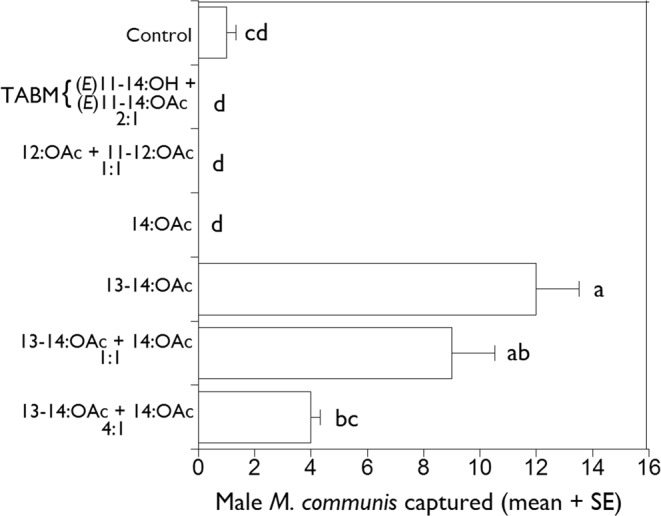
Mean total capture ( + SE) per trap of male *Melanotus communis* beetles in traps baited with 12-carbon and 14-carbon acetates in 2016 (4 August – 13 September). Means with the same letter are not significantly different (P > 0.05).

In preliminary bioassays in 2016, a total of 26 male *M. communis* were caught, with lures containing 13–14:OAc being most attractive (Fig. 3). In particular, 13–14:OAc as a single component attracted marginally more beetles (MRPP; P = 0.053, A = 0.081) than the 4:1 binary blend of 13–14:OAc + 14:OAc, but not more (P > 0.05) than the 1:1 blend of these two compounds. Captures in the 1:1 and 4:1 blends of 13–14:OAc + 14:OAc were not significantly different (P > 0.05). The 4:1 blend of 13–14:OAc + 14:OAc did not differ (P > 0.05) from the control. No beetles were caught in the traps baited with 14:OAc, the 12:OAc + 11–12:OAc blend, or TABM pheromone.

**Figure 4:**
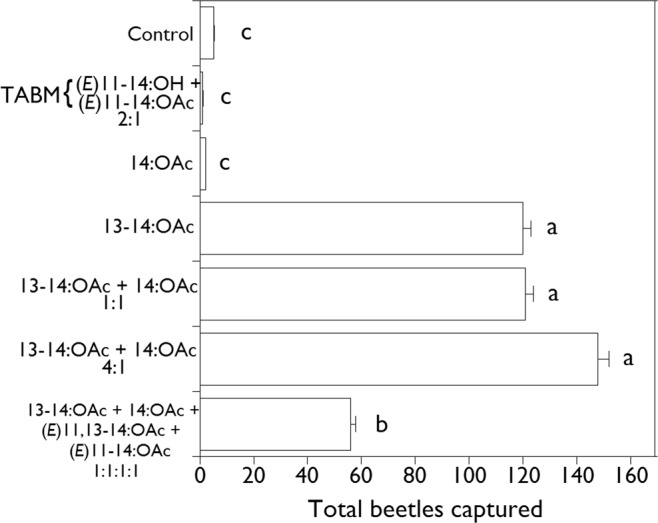
Mean total capture ( + SE) per trap of male *Melanotus communis* beetles in traps baited with 14-carbon acetates in 2017. Means with the same letter are not significantly different (P > 0.05).

In 2017, using plastic bag lures and Vernon Pitfall Traps^®^ instead of plastic tube dispensers and sticky traps, 449 male *M. communis* were attracted to treatments containing the 14-carbon acetates, and trends were similar to those of 2016. The 4:1 and 1:1 blends of 13–14:OAc + 14:OAc and 13–14:OAc as a single component were not significantly different (Fig. 4), whereas all of these were significantly more attractive than the 1:1:1:1 blend of 13–14:OAc + 14:OAc + (*E*)-11,13–14:OAc + (*E*)-11-14:OAc (Fig. 4). The TABM pheromone, 14:OAc, and control treatments attracted one, two, and five males, respectively, and did not differ from each other (P > 0.05).

### Dose-response tests with 13-tetradecenyl acetate

**Figure 5:**
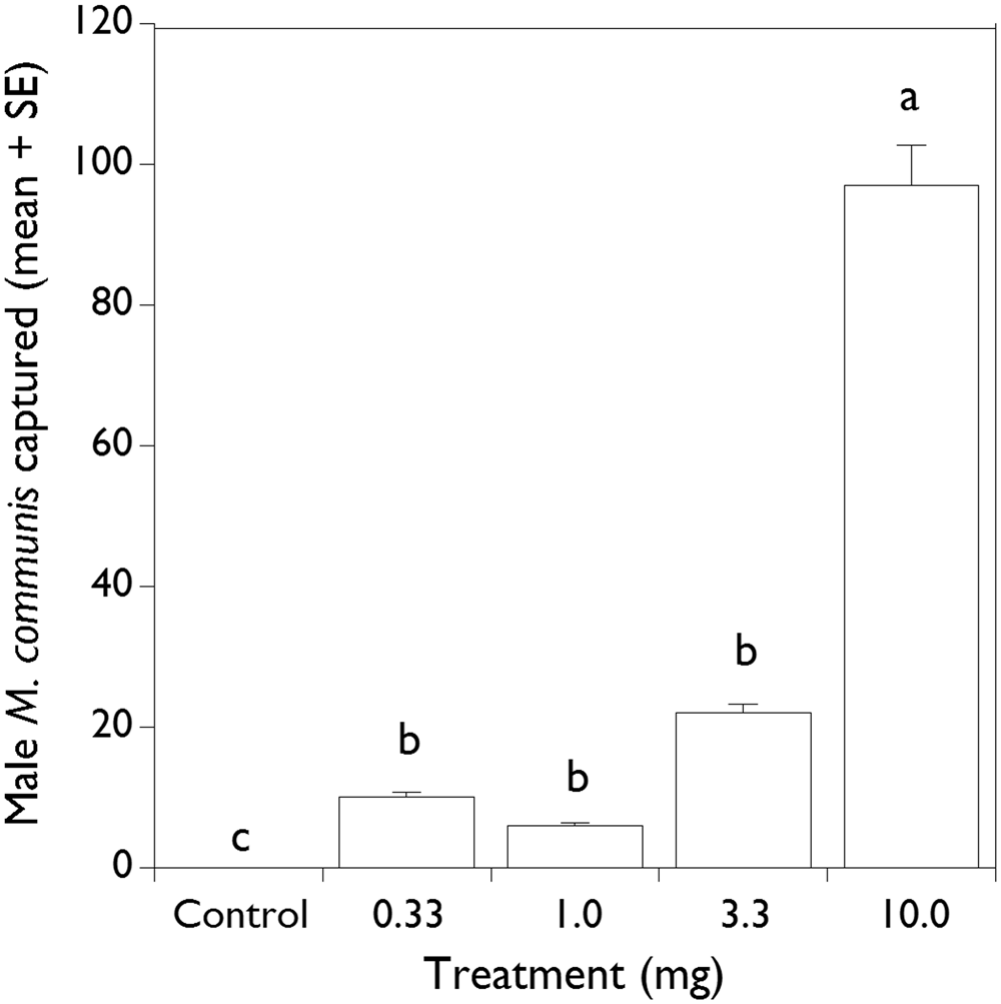
Mean total capture ( + SE) per trap of male *Melanotus communis* beetles in traps baited with 0–10 mg doses over a semi-log range in 2018. Means with the same letter are not significantly different (P > 0.05).

In the 2018 trial using a semi-log range of doses, a total of 135 male *M. communis* were captured. The 10 mg dose was significantly more attractive than the lower doses (Fig. 5; MRPP 10 vs 0.33; P = 0.002, A = 0.318) (MRPP 10 vs 1; P = 0.001, A = 0.401) (MRPP 10 vs 3.3; P = 0.018, A = 0.165). Captures with the 0.33, 1.0, and 3.3 mg doses were not significantly different from each other (P > 0.05), but were significantly greater than the control.

### Comparison of pheromone dispensers for 13-tetradecenyl acetate

**Figure 6:**
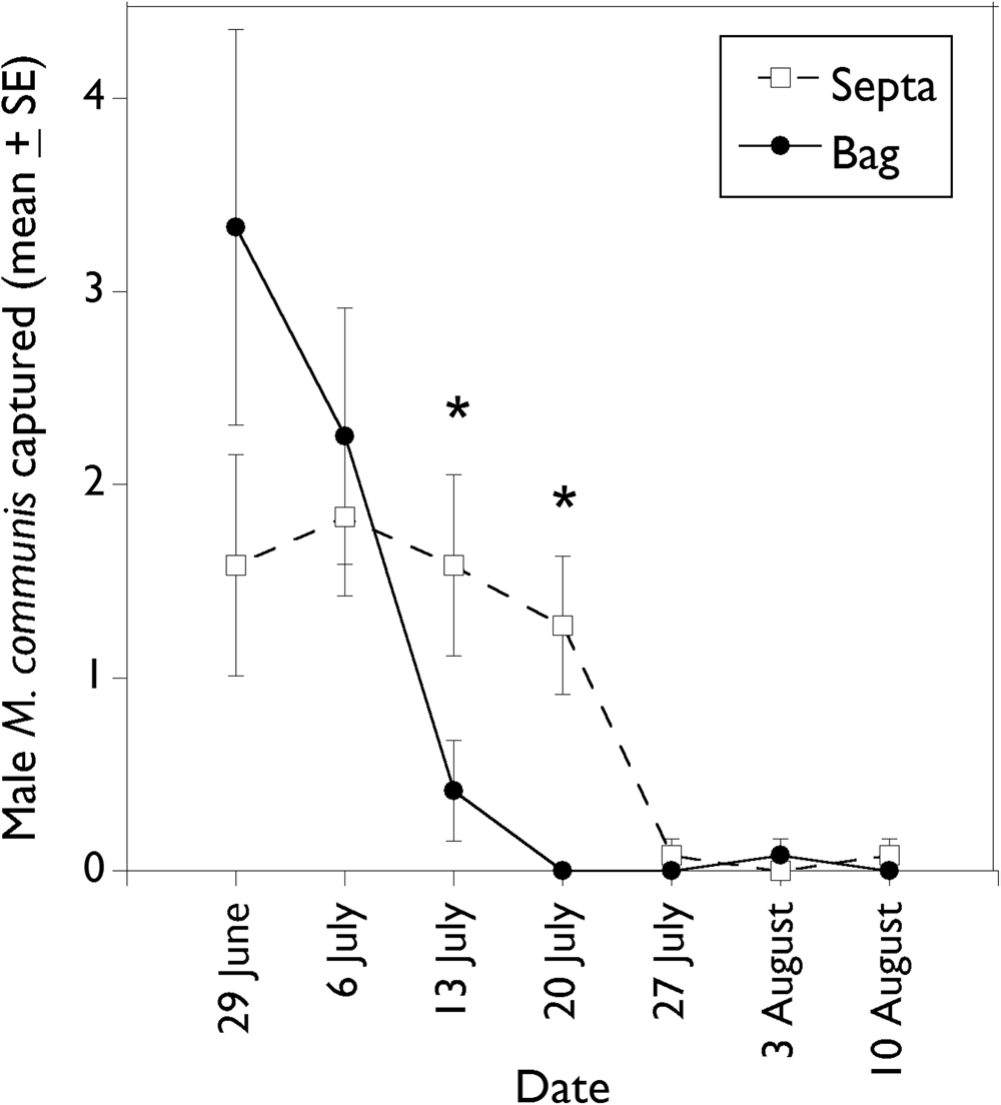
Weekly capture (mean ± SE) per trap of male *Melanotus communis* beetles in traps baited with 10 mg 13–14:OAc released from rubber septa or polyethylene bags in 2018. Dates with an asterisk indicate significant treatment effect (P < 0.05).

In this trial comparing equal doses of pheromone released from plastic bag or rubber septum dispensers, a total of 149 male *M. communis* were captured (septa = 76; bags = 73). Captures in traps baited with the polyethylene bags declined in an exponential manner from 3.33 males per trap in wk 1 (22–29 June) to zero in wk 4 (13–20 July), after which trap catches were negligible (Fig. 6). Captures in traps baited with the rubber septa were relatively stable, i.e., ca. 1.6 males per trap per week for 4 wk (22 June–20 July), after which trap catches declined (Fig. 6). For both dispenser types, trap catches after wk 4 were at or near zero. Catches in traps baited with plastic bags were not significantly different (P > 0.05) than those in traps baited with septa at wk 1 and 2 (22–29 June and 29 June – 6 July), but were significantly less than the septa in wk 3 (6–13 July; T_11_ = 4.00, P = 0.0021) and wk 4 (13–20 July; T_10_ = 3.78, P = 0.0036).

### Diversity of *Melanotus* species at the field sites and specificity of pheromone lures

Of the 758 *Melanotus* individuals captured in traps baited with test chemicals during the pheromone field bioassays, all but two were male *M. communis*. The exceptions were one male *M. lanei* Quate (1–8 May 2017) and one female *M. piceatus* Blatchley (8–15 May 2017), both of which were captured in traps baited with the 1:1 mixture of 13–14:OAc + 14:OAc. An additional 85 *Melanotus* individuals were captured in Malaise traps, 43 of which were *M. communis*. The remaining *Melanotus*, and their numbers, captured in the Malaise traps were: *M. piceatus* (15), *M. verberans* (15)*, M. corticinus* (Say) (5), *M. morosus* Candèze (3)*, M. similis* (3), and *M. americanus* (Herbst) (1), but no males of any of these species were caught in any of the lure-baited traps, indicating a high species specificity of the *M. communis* pheromone.

## Discussion

Our analytical and bioassay data indicate that 13–14:OAc is the major and likely only component of the female-produced sex pheromone of *M. communis*. Male *M. communis* were not attracted to lures that did not have 13–14:OAc as a component. The analog 14:OAc, although present in volatiles collected from crushed abdomens of females, appeared to be inactive, neither increasing or decreasing attraction of males. In addition, 14:OAc elicited minimal responses from antennae of males in GC-EAD bioassays, further suggesting that it is not a crucial component of the pheromone. Thus, 14:OAc may have a role as an inhibitory compound preventing cross-attraction of congeners that might also use 13–14:OAc as a pheromone component, or it may simply be present as a biosynthetic precursor to the pheromone or even an artifact. Our analyses were based on volatiles collected from crushed abdomens rather than the volatiles actually released by calling females, because none of the live females emitted detectable amounts of these or related compounds under laboratory conditions.

In tests of analogs of 13–14:OAc, the TABM pheromone blend was not attractive to *M. communis*, and *E*11-14:OAc elicited no responses from antennae of males in GC-EAD assays. In contrast, *E*11, 13–14:OAc did elicit EAD responses, but if anything, the field bioassay data from 2017 suggested that it might be inhibitory (Fig. 4). Thus, the EAD responses seen may have been a result of the structural similarity between *E*11, 13–14:OAc and the actual pheromone, 13–14:OAc.

In our initial trials in which test pheromone blends were deployed in plastic PCR tubes with a pinhole in the cap, only low numbers of beetles were caught, suggesting that the release rate of these dispensers might be too low. This result was corroborated in subsequent trials in which either small plastic bags or rubber septum dispensers loaded with similar doses of pheromone attracted substantially more male beetles than the PCR tube dispensers. A dose-response bioassay testing a semi-log range of doses showed that the 10 mg dose, dispensed from plastic bags, was significantly more attractive than any of the lower doses. In a follow-up field trial, the plastic bag dispensers were compared to the rubber septa which have found widespread use as pheromone dispensers for lepidopteran species, with both dispensers loaded with 10 mg of pheromone. Under typical summer climatic conditions, the septa appeared to have longer and more consistent field lifetimes than the polyethylene bags. Specifically, the two dispenser types attracted similar numbers of beetles for the first two weeks after deployment, after which the attractiveness of the plastic bags appeared to decrease more quickly than that of the rubber septa. Five weeks after deployment (i.e., late July), both lure types attracted minimal numbers of beetles, but this may have been due to the natural seasonal decline in *M. communis* reproductive activity.

During our field trials, conducted over several years with a number of potential pheromone candidates for *Melanotus* species, we saw no evidence of cross attraction of any other elaterid species to 13–14:OAc, nor did we see any evidence of attraction to any of the analogs or homologs that were tested. These data indicate that 13–14:OAc may be species-specific to *M. communis*, especially because a number of other *Melanotus* species were detected at our field sites using other monitoring methods. In fact, it was surprising that no other species were attracted to any of these compounds or blends given that many closely related elaterids have the same or similar pheromone components^1,2,39–41^. In sum, these results suggest that the pheromone components or pheromone blends of *Melanotus* and other click beetle species may be both quite diverse, but also narrowly tuned, with each species using either relatively unique compounds, or very specific ratios of compounds that may be shared by more than one species.

Our study is the first identification of a sex pheromone for a North American *Melanotus* species. Of the two other species in the genus for which pheromones have been fully reported, both from Japan, both produce esters of 12-carbon saturated and unsaturated alcohols. Thus, dodecyl acetate (12:OAc) was identified as the single sex pheromone component of *M. okinawensis*^23,42^, whereas a blend of (*E*)-9,11-dodecadienyl butanoate and (*E*)-9,11-dodecadienyl hexanoate were identified as sex pheromone components of *M. sakishimensis*^24,42^. (*E*)-9,11-Dodecadienyl butanoate and (*E*)-9,11-dodecadienyl hexanoate have also been identified in extracts of *M. tamsuyensis*, although bioassay data have not been reported^25^. Published observations in Europe suggest that *M. punctolineatus* (Pelerin) may use a tetradecenyl butyrate as a pheromone, whereas *M. rufipes* (Herbst) may use a tetradecadienyl butyrate^43^. Pheromone glands of the west-Eurasian *M. fusciceps* and the Holarctic *M. castanipes* contained mixtures of > 22 compounds, which included esters of 12-carbon and 14-carbon saturated and unsaturated alcohols, although the exact structures were not identified, nor were any bioassay data reported^22^. Thus, the compounds that we identified from *M. communis* in the present study are clearly similar to the pheromones or pheromone candidates found in other *Melanotus* species from Japan and Europe, especially the 14-carbon compounds of the west-Eurasian beetles. Further studies of the sex pheromones of additional *Melanotus* species should provide a more complete picture of the chemical diversity of the sex pheromones within this taxonomically large genus.

Insect sex pheromones have been exploited for pest management for more than 50 years, including the pheromones of click beetles. In Europe, pheromone-based methods have been particularly valuable for monitoring pest species^44–46^, as well as threatened non-pest species^47^. Pheromone-based methods also have been used to monitor several invasive Eurasian click beetle species, i.e., *Agriotes* spp., that are now established in Canada and the U.S.^48^. In addition, sex pheromones were exploited in management of pest species, including mass trapping^20^ and mating disruption^21^ of *M. okinawensis*, and may have potential in attract-and-kill strategies using entomopathogens^49^. Given that the pheromone of *M. communis* consists of a single component that can be readily synthesized, development of its pheromone for monitoring and control purposes may be both possible and economically feasible.

### Ethical approval

The laboratory research was conducted at the University of California, Riverside, and field bioassays were conducted at the USDA-ARS U.S. Vegetable Laboratory and Clemson University Coastal Research and Extension Center in Charleston. All methods met the ethical requirements of the respective university and the United States Department of Agriculture, and followed guidelines of the Committee of Publication Ethics. This article does not involve any studies with human participants or vertebrate animals.

## Data Availability

The datasets generated during and/or analyzed during the current study are available from the corresponding author on reasonable request.
